# NPBS database: a chemical data resource with relational data between natural products and biological sources

**DOI:** 10.1093/database/baaa102

**Published:** 2020-12-11

**Authors:** Tingjun Xu, Weiming Chen, Junhong Zhou, Jingfang Dai, Yingyong Li, Yingli Zhao

**Affiliations:** Shanghai Institute of Organic Chemistry, Chinese Academy of Sciences, 345 LingLing Road, Shanghai 200032, China; Shanghai Institute of Organic Chemistry, Chinese Academy of Sciences, 345 LingLing Road, Shanghai 200032, China; Shanghai Institute of Organic Chemistry, Chinese Academy of Sciences, 345 LingLing Road, Shanghai 200032, China; Shanghai Institute of Organic Chemistry, Chinese Academy of Sciences, 345 LingLing Road, Shanghai 200032, China; Shanghai Institute of Organic Chemistry, Chinese Academy of Sciences, 345 LingLing Road, Shanghai 200032, China; Shanghai Institute of Organic Chemistry, Chinese Academy of Sciences, 345 LingLing Road, Shanghai 200032, China

## Abstract

NPBS (Natural Products & Biological Sources) database is a chemical data resource with relational data between natural products and biological sources, manually curated from literatures of natural product researches. The relational data link a specific species and all the natural products derived from it and contrarily link a specific natural product and all the biological sources. The biological sources cover diverse species of plant, bacterial, fungal and marine organisms; the natural molecules have proper chemical structure data and computable molecular properties and all the relational data have corresponding references. NPBS database provides a wider choice of biological sources and can be used for dereplication to prevent re-isolation and re-characterization of already known natural products.

**Database URL**: http://www.organchem.csdb.cn/scdb/NPBS

## Introduction

The studies of deriving natural products from biological sources presented in publications provide abundant information of diversity in both biology and chemistry. Although knowledge of natural products has inspired new medicines, agrochemicals and materials, broad research on universal natural products derived from various organisms can be deficient (1–3). For example, the correlations between the molecules of natural products and their host species are still ambiguous. What are the already known chemical components or metabolites of one specific species? What are the relationships between species sharing identical chemical components or metabolites? Those mysteries may be studied by computational approaches, which condensed the widespread information from literatures and combined them logically for further applications. There are already several data resources useful for natural product researches (Table 1). The data resources are various from free access and commercial, from comprehensive and specialized, nevertheless, there are lack of available datasets that have clarity about the relationships between natural products and biological sources, as natural product deriving achievements are increasingly published in journals (13–16).

**Table 1.** Existing data resources of natural product researches

Name	Availability	References
Dictionary of Natural Products (DNP)	http://dnp.chemnetbase.com	
Reaxys	https://www.reaxys.com	
Super Natural II	http://bioinf-applied.charite.de/supernatural_new	4
Universal Natural Products Database (UNPD)	http://pkuxxj.pku.edu.cn/UNPD	
TCM database@ Taiwan	http://tcm.cmu.edu.tw	5
TCMID	http://www.megabionet.org/tcmid	6
Chem-TCM	http://www.chemtcm.com	7
NuBBE database	http://nubbe.iq.unesp.br/portal/nubbedb.html	8
Dictionary of Marine Natural Products (DMNP)	http://dmnp.chemnetbase.com	
AntiBase	http://wwwuser.gwdg.de/∼hlaatsc/antibase.htm	
MarinLit	http://pubs.rsc.org/marinlit	
TIPdb	http://cwtung.kmu.edu.tw/tipdb	9
NPCARE	http://silver.sejong.ac.kr/npcare	10
NANPDB	http://www.african-compounds.org/nanpdb	11
StreptomeDB	http://www.pharmaceutical-bioinformatics.de/streptomedb	12

Data collections of chemical information (molecular structures, syntheses and reactivities, chemical and biological properties, etc.) are essential in chemical research. The databases mentioned above may be focused on a special group of chemical compounds (natural products, marine compounds, plant chemicals, etc.). The main feature of these databases is, however, that the search result is the chemical information of a natural product when given a query of molecular structure or documents of a reporting natural product when given a query of biological species name. The search results could be direct information of biological sources when the search is a natural product or information of natural products when search is a biological species name, if the relationships between natural products and biological sources have been established. We herein describe a chemical data resource, NPBS (Natural Products & Biological Sources) database, which includes the information of relationships between natural products and biological sources reported in publications. The relational data link a specific species and all the natural products derived from it and contrarily link a specific natural product and all the biological sources, and each relational data has corresponding references. In this database, the natural products are represented by the molecular structures of the molecules derived from organisms, and the biological sources are represented by the species names of the organisms. Other available information like deriving parts of the organisms, names of the natural products and computable molecular properties are also included in the NPBS database. The volume of the database is extending continuously, as journal literatures of natural product–deriving researches are on the increase, and we intend to involve more publications in the data acquisition stage.

## Materials and methods

**Figure 1.**
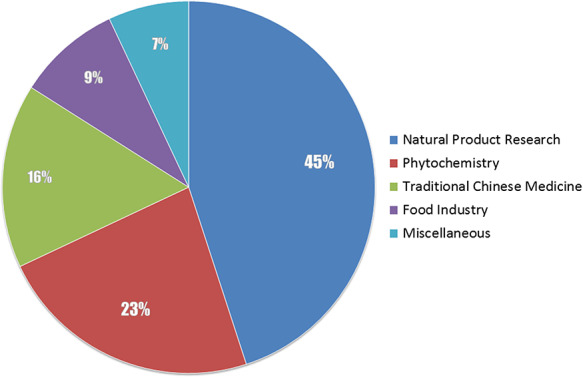
The coverage of the journals involved in the NPBS database.

The core idea of this work is to link natural products and biological sources, and our priority is obtaining such information of relationships. We then focused on scientific literatures, seeing that literatures of natural product–deriving researches invariably report biological species from which they extract the natural molecules. As a strategy of screening literatures for data sources, our data analysts browse the contents of each journal by issues and volumes, select the required articles based on rough judgments of the titles and abstracts and obtain the full text PDF version of the articles, or abstract texts and bibliographic information when the PDF file is unavailable. The collected literatures would be screened again by inspecting the main body of the articles before extracting data from them, to exclude literatures that have no descriptions of natural products and biological sources. We involved the major publications of natural product–deriving researches both domestic (Chinese) and international (English), 45% of them are extensive research on natural products, others are on phytochemistry, traditional Chinese medicine, food industry and miscellaneous, as shown in Figure 1. Classification of a selected journal is based on the scope description of the journal. The main list of publications that NPBS database covered is provided as Supplemental data.

**Figure 2.**
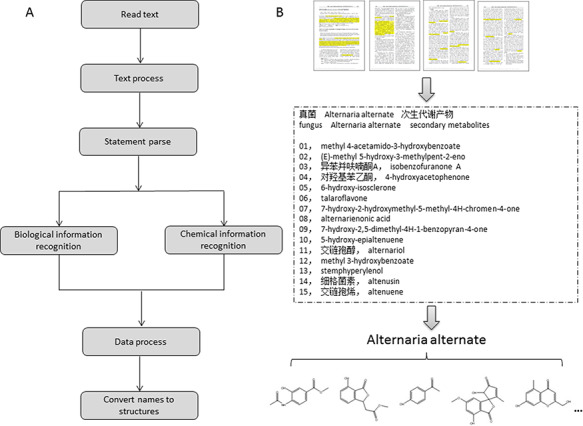
The design of the text-mining system. (A) Flow process of the data text-mining system. (B) Demonstration of the procedure for data acquisition.

In the early stage of this project, we collected the raw data of NPBS database from publications manually. Our data analysts reviewed the literatures and indexed the information of biological sources and the molecules of natural products. With the practical experience and plenty of hand-curated data, we developed a text-mining system (NPBS_sys_) for computer-aided data acquisition and attempted to extract the required data information from the textual description of the journal literatures automatically (Figure 2). The NPBS_sys_ used the technology of named entity recognition (NER); more specifically, a methodology of NER based on rules and dictionaries was implemented in this system (17). The biological information of the species names and parts was matched by dictionaries we built, for example, ‘*Salvia kiaometiensis*’ and ‘roots’ in the sentence of ‘study the chemical constituents of the roots of *Salvia kiaometiensis*’. The chemical names of natural products were matched by rule templates we designed, for example, ‘euscaphic acid’ and ‘hyptadienic acid’ in the sentence of ‘two compounds were obtained and identified as euscaphic acid and hyptadienic acid’.

The biological information recognized in NPBS_sys_ is the species description of the organism, such as ‘endophytic fungus *Alternaria alternata*’ in Figure 2B (18), and deriving parts of the organisms, such as ‘secondary metabolites’, ‘leaves’ and ‘aerial parts’. The chemical information recognized in NPBS_sys_ is trivial, systematic or semi-systematic names of the natural product molecules and the author numbers of the molecules. The author numbers such as ‘Compound A’ and ‘Compound (1)’, are used for associating different representations of the same natural product appear in abstract, introduction, results and experimental section, or English and Chinese names of the same natural product in Chinese literatures. When the data meet the definition of ‘large scale’, that information will be complementary in natural product name translation and molecular structure converting. As an exploration of chemical text-mining, the NPBS_sys_ not only get the chemical entities in literatures but also take an attempt to recognize the connections between the entities. For example, the relationship of natural product ‘β-carboline alkaloid’ and biological source ‘*Carthamus tinctorius*’ recognized in the sentence ‘A new β-carboline alkaloid was isolated from the leaves of *Carthamus tinctorius*’ is the connection between these two entities. Nevertheless, a great quantity of literatures are not available as text-mining materials, and some literatures do not provide appropriate systematic names for the ‘new-found’ natural product molecules, leading to extra procedure of hand-drawing structures or web searching. Therefore, most available data in NPBS database rely on manual work at present.

In the process of NPBS_sys_, the structures of the molecules are generated by ‘name to structure module’ of ACDLabs (http://www.acdlabs.com), ChemOffice (http://www.cambridgesoft.com) and OPSIN (19), and we use ChemDraw (http://www.cambridgesoft.com) and Reaxys (https://www.reaxys.com) for drawing and searching the structures manually. A machine translation tool of chemical nomenclature has been used for Chinese compound names to English translation (http://www.organchem.csdb.cn/translate). We have an evaluation of the structures generated by different toolkits, scores have been made by molecular formula comparison in order to evaluate the different structures from the same compound name, and the eventual structures are standardized to MDL Molfile format. RDKit (http://www.rdkit.org) has been applied in Python (https://www.python.org/) for molecular properties computing in NPBS database.

The raw data had been processed properly before added into NPBS database. We first assume the correctness of the primary literatures, unless there are apparent errors like typos, and then backtrack on the original document when encounter abnormal data in the subsequent processing. For the same biological source from multiple literatures, we merge the data and remain the distinct natural products and list all the references. We have similar approach for the same natural product derived from different biological sources reported by multiple literatures. When encounter multiple biological sources in one literature, for example, components of mixed species researches (20), we label the relational data as ‘unconfirmed’; once other literatures reported that data corroborated the natural products from one of those biological sources, the relational data will be ‘confirmed’.

## Results and discussion

**Figure 3.**
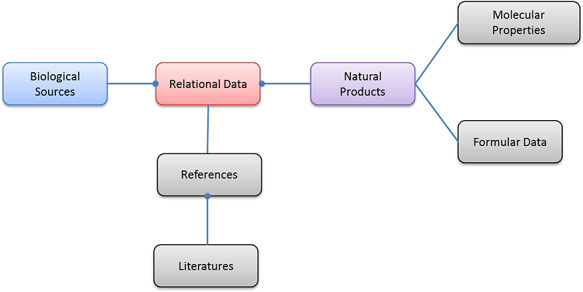
The entity relationship diagram of NPBS database.

At the time of writing, there are 33 377 unique biological sources (distinguished by species names), 122 776 unique molecules of natural products (distinguished by InChIKey) (21) and 898 294 relational data records included in NPBS database. The biological sources cover the diverse species of plant, bacterial, fungal and marine organisms, the molecules have proper chemical structure data and computable molecular properties, and all the relational data have corresponding references. The entity relationship diagram of NPBS database is shown in Figure 3; other features of the current database are shown in the following tables and figures.

The top 10 species of biological sources that provide most natural products in NPBS database, as shown in Table 2, are all plants as expected. On one hand, literatures of phytochemistry are the majority of the publications we covered at present. On the other hand, terrestrial plants are the most abundant and accessible biological source on the earth, and human beings have a long history of taking plants as food, medicines and materials. Interestingly, the top two and the ninth species are fruits, other five species are used as seasoning and spice, each one provide over 700 natural products. Two traditional Chinese herbal medicines *Artemisia annua* and *Hypericum perforatum* have been demonstrated by modern science; their special constituents show significant antimalarial and antidepressant activity (22, 23).

**Table 2.** The top 10 species of biological sources provide most natural products

			Number
			of natural
No.	Species name	Common name	products
1	*Mangifera indica*	Mango	1152
2	*Vitis vinifera*	Grape	846
3	*Rosmarinus officinalis*	Rosemary	790
4	*Artemisia annua*	Sweet sagewort	797
5	*Capsicum annuum*	Cayenne pepper	764
6	*Ocimum basilicum*	Sweet basil	761
7	*Hypericum perforatum*	Common St. John’s wort	731
8	*Foeniculum vulgare*	Sweet fennel	724
9	*Psidium guajava*	Guava	723
10	*Coriandrum sativum*	Coriander	723

The top 10 molecules of natural products derived from most biological sources in NPBS database, as shown in Table 3, are 8 terpenoids, 1 steroid and 1 aliphatic acid, each one is derived from over 4000 biological sources. Terpenoids are a large group of substances, which occur in most organisms, playing vital roles of biofunctionality such as antioxidants and nutritions (24). The steroid ‘β-sitosterol’ and the aliphatic acid ‘palmitic acid’ also widely exist in organisms as important classes of bioorganic molecules (25).

**Table 3.** The top 10 molecules of natural products derived from most biological sources

No.	Molecule	Name	Number of bio- logical sources
1	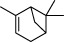	α-pinene	6609
2	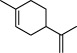	limonene	6064
3	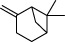	β-pinene	5899
4	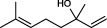	(-)-β-linalool	5730
5	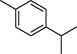	p-cymene	5354
6	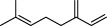	myrcene	5304
7	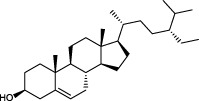	β-sitosterol	5112
8	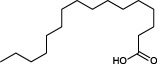	palmitic acid	4871
9	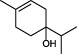	(-)-terpinen-4-ol	4749
10	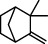	camphene	4636

**Figure 4.**
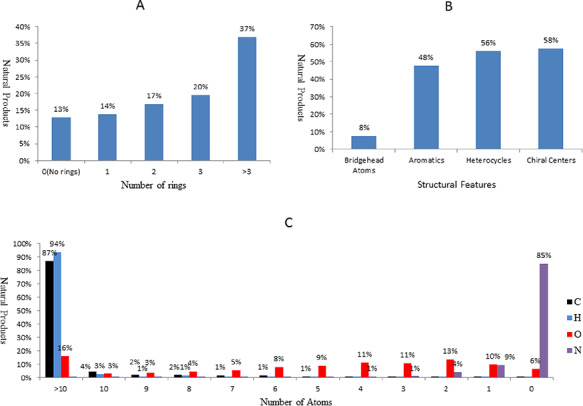
The molecular features of the natural products in NPBS database. (A) Number of rings. (B) Structural features. (C) Number of atoms.

The molecular features of the natural products in NPBS database, as shown in Figure 4, are perceived as chemically different from the molecules in other chemical databases (http://www.organchem.csdb.cn/). For the structural complexity, >86% of the natural products have ring system, over one-third have more than three rings (Figure 4A), and 56% of them are heterocycles (Figure 4B). Approximately half of the natural products are aliphatic, 58% of them have chiral centers (Figure 4B). The natural products also present extremely higher oxygen content on average, over 93% of them have oxygen atom, and the percentage of the natural products having >10 oxygen atoms reach 16%; it seems odd when compared with nitrogen content (Figure 4C).

**Figure 5.**
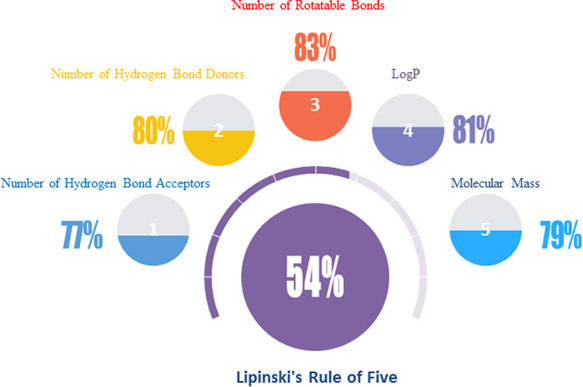
The ‘drug-likeness’ features of the natural products in NPBS database.

For the interest of taking natural products as starting points for medicinal chemistry and drug discovery, the Lipinski’s rule of five parameters may have significant referential value and have insight into ‘drug-likeness’ of the molecules in NPBS database (26). Over half of the natural products are within the bounds of Lipinski’s primary five parameters (Figure 5): molecular weight <500, number of hydrogen bond donors <5, number of hydrogen bond acceptors <10, number of rotatable bonds <10 and LogP <5. There is no doubt that the natural products are the treasure of potential drug candidates.

For evaluation of NPBS database, we carried out an experiment of comparison with other accessible natural product databases by searching for several common natural products and biological sources. The result as listed in Table 4 shows that NPBS database may be more applicable for searching wide biological sources of a specific natural product and molecular properties of natural products derived from a specific biological source. For example, NPBS database shows 17 biological sources that contain natural product of ‘aconitine’ and 49 natural products derived from biological source of ‘*Artemisia apiacea*’.

**Table 4.** Comparison with NPBS and other accessible natural product databases

Data resource	Result of search-ing natural products	Result of searching biological sources
Super Natural II	Molecular properties	Not available
TCM Database	Molecular properties	Only some of Chinese Medicinal Herbs available
Reaxys	Substances and documents	Documents
TCMID	Not available	Only some of Traditional Chinese Medicines available
NuBBE database	Number of results	Number of results
TIPdb	No result	No result
NPBS	Molecular properties and biological sources	Natural products and molecular properties

## Conclusions

Compared with the top databases of natural product (Table 1), NPBS database has not exceed their volume or coverage, but has greater clarity about the relationships between natural products and biological sources. With continuous upgrading and optimizing of the text-mining system, we might have an efficient and low-cost tool to expand the volume of NPBS database in the future. However, we have not concentrated on the classification and analysis of the biological source data. Nevertheless, current datasets in NPBS database have shown considerable diversity both in biology and chemistry, and the relational data provide significant clues in the correlations between some special natural products and their host species.

Besides mining information from literatures, to make published data quickly accessible and to combine them logically for further applications, the main purpose or destination of this database is clarifying about the relationships between natural products and biological sources, since the relationships are intersecting. For example, the same natural product and its homologues may originate from various biological sources; adopters can have a wider choice on biological sources from their own concerns. On the other hand, integrated constituent information from a specific biological source can inspire novel applications of the biological source, for example, adopters can analyze the potential toxicology or pharmacology of a Chinese medicinal herb at the molecular level. Adopters can also use the natural product structures from a specific biological source for dereplication with experimental data, to prevent re-isolation and re-characterization of already known molecules. We are looking forward to discover more secrets of natural products using new approach of cheminformatics and providing sophisticated data support for pharmaceutical research by web interface and retrieval functionality.

## Supplementary Material

baaa102_Supp
