# Supplementary material for: NPBS database: a chemical data resource with relational data between natural products and biological sources
Source: Database (Oxford). 2020 Dec 11;2020:baaa102. doi: 10.1093/database/baaa102 (PMC7731925; doi:10.1093/database/baaa102)
Supplement: baaa102_Supp [file baaa102_supp.zip › Coverage.pdf]

Main list of publications:

Chemistry of Natural Compounds

J. Nat. Prod.

Journal of Agricultural and Food Chemistry

Natural Product Research

Fitoterapia

Zhongcaoyao

Journal of Asian Natural Products Research

Phytochemistry

Asian Journal of Chemistry

Natural Product Communications

Shipin Gongye Keji

Tianran Chanwu Yanjiu Yu Kaifa

Food Chemistry

Shipin Kexue (Beijing, China)

Biochemical Systematics and Ecology

Zhongguo Zhongyao Zazhi

Molecules

Planta Med.

Journal of Ethnopharmacology

Zhongyaocai

Industrial Crops and Products

Shizhen Guoyi Guoyao

Food and Chemical Toxicology

Helvetica Chimica Acta

Xiandai Shipin Keji

Journal of Essential Oil Research

Bioorganic & Medicinal Chemistry Letters

Journal of Separation Science

J. Sci. Food Agric.

Phytochemical Analysis

Chem. Pharm. Bull.

Tetrahedron

Chemistry & Biodiversity

Organic Letters

Archives of Pharmacal Research

Zhongchengyao

J. Chem. Ecol.

J. Antibiot.

Journal of Natural Medicines

Quimica Nova

Journal of Pharmaceutical and Biomedical Analysis

Yancao Keji

Pharmaceutical Biology (London, United Kingdom)

Zhongguo Yaoxue Zazhi (Beijing, China)

Yaowu Fenxi Zazhi

Natural Medicines (Tokyo, Japan)

Shenyang Yaoke Daxue Xuebao

Yiyao Daobao

Zhongguo Tianran Yaowu

Guangdong Yaoxueyuan Xuebao

Zhongguo Tiaoweipin

Zhongguo Yiyuan Yaoxue Zazhi

Zhongguo Yaoye

Heterocycles

Grasas y Aceites (Sevilla, Spain)

Journal of the Brazilian Chemical Society

Zeitschrift fuer Naturforschung, C: Journal of Biosciences

Lihua Jianyan, Huaxue Fence

Bulletin of the Korean Chemical Society

J. Food Sci.

Steroids

Zhongguo Liangyou Xuebao

Shipin Keji

Zhongguo Xiandai Zhongyao

Guangpu Shiyanshi

Zhongguo Youzhi

Carbohydr. Res.

Biosci., Biotechnol., Biochem.

Zhongguo Haiyang Yaowu

Phytomedicine

Pharmaceutical Chemistry Journal

Biological & Pharmaceutical Bulletin

South African Journal of Botany

Bioorganic & Medicinal Chemistry

Magnetic Resonance in Chemistry

Huaxi Yaoxue Zazhi

Zhongguo Xiandai Yingyong Yaoxue

Zhongguo Yiyao Gongye Zazhi

Guangxi Zhiwu

Weishengwu Xuebao

Chinese Chemical Letters

Yaoxue Xuebao

Shipin Yu Fajiao Gongye

Journal of Oleo Science

Zhongguo Shipin Tianjiaji

Indian Drugs

Fenxi Shiyanshi

Redai Yaredai Zhiwu Xuebao

Huaxue Yanjiu Yu Yingyong

Xiangliao Xiangjing Huazhuangpin

Yunnan Daxue Xuebao, Ziran Kexueban

Sepu

Lipids

Z. Lebensm.-Unters. Forsch.

Jingxi Huagong

European Journal of Lipid Science and Technology

Tetrahedron Lett.

Journal of the American Oil Chemists' Society

Yingyang Xuebao

Linchan Huaxue Yu Gongye

Chinese Journal of Chemistry

Oriental Journal of Chemistry

Fenxi Huaxue

Beijing Gongshang Daxue Xuebao, Ziran Kexueban

Fenxi Ceshi Xuebao

Zhongguo Yaoke Daxue Xuebao

Journal of Chemical Research

Zhongguo Yaowu Huaxue Zazhi

Siliao Yanjiu

Z. Naturforsch., C: Biosci.

Flavour and Fragrance Journal

J. Food Sci. Technol.

Zhongshan Daxue Xuebao, Ziran Kexueban

Zeitschrift fuer Naturforschung, B: Chemical Sciences

Sci. Pharm.

Indian J. Chem., Sect. B

Zhipu Xuebao

Ziyuan Kaifa Yu Shichang

Linye Kexue

European Journal of Organic Chemistry

Plant Foods Hum. Nutr. (Dordrecht, Neth.)

Youji Huaxue

Beijing Zhongyiyao Daxue Xuebao

Journal of Saudi Chemical Society

Yuanyi Xuebao

Can. J. Chem.

Planta

Journal of Supercritical Fluids

Natl. Acad. Sci. Lett. (India)

Xibei Zhiwu Xuebao

Huaxue Xuebao

Journal of Food Composition and Analysis

Bopuxue Zazhi

Yunnan Zhiwu Yanjiu

Xinxiang Yixueyuan Xuebao

Zhongguo Shouyi Zazhi

Zhiwu Yichuan Ziyuan Xuebao

Plant Physiol.

Zhiwu Xuebao

Chromatographia

Comptes Rendus Chimie

J. Indian Chem. Soc.

J. Chin. Chem. Soc. (Taipei)

Fujian Shifan Daxue Xuebao, Ziran Kexueban

Aust. J. Chem.

Guangdong Gongye Daxue Xuebao

Nanhua Daxue Xuebao, Yixueban

Keji Daobao

Zhongguo Keji Lunwen Zaixian

Huaxue Tongbao

Xuzhou Yixueyuan Xuebao

Chengshi Huanjing Yu Chengshi Shengtai

Lanzhou Daxue Xuebao, Ziran Kexueban

Hebei Keji Shifan Xueyuan Xuebao

Xibei Shifan Daxue Xuebao, Ziran Kexueban

Journal of Chinese Pharmaceutical Sciences

Turkish Journal of Chemistry

J. Org. Chem.

Daru, Journal of Faculty of Pharmacy, Tehran University of Medical Sciences

Chem. Lett.

European Food Research and Technology

Huaqiao Daxue Xuebao, Ziran Kexueban
